# Hospital and Institutional News

**Published:** 1920-10-16

**Authors:** 


### HOSPITAL AND INSTITUTIONAL NEWS.

### THEIR MAJESTIES AND "OUR DAY."

The King sent the following message to the British Red Cross Society and the Order of St. John of Jerusalem: "I am glad to know that the British Red Cross Society and the Order of St. John of Jerusalem are continuing to work together in peace time as in war, and that they have decided again to hold 'Our Day' in aid of the funds of the Joint Council. I realise the unceasing demands made upon its resources on behalf of the sick and suffering, and as patron of King Edward's Hospital Fund for London I view with grave anxiety the position of voluntary hospitals, both in the Metropolis and throughout the country. I wish every success to your efforts. By the dissemination of knowledge the Joint Council are wisely directing attention not only to the curing, but to the prevention of sickness and disease, while the combined action of the two societies marks an important step towards the co-ordination of voluntary work.—George, R.I."

### THE QUEEN'S MESSAGE.

Queen Mary sent the following message: "It gives me great pleasure to hear that it has been decided to revive 'Our Day' in aid of the funds of the Joint Council of the British Red Cross Society and the Order of. St. John of Jerusalem. I have followed with keen interest the good results which have attended the labours of the Joint Council in times of war, and I earnestly hope and pray that their renewed efforts in times of peace may meet with a response that will enable them to achieve an equal or even greater measure of success.—Mary, R."

### THE PRINCE OF WALES.

The following message from the Prince of Wales was received: "I have just heard on my return to London that 'Our Day,' in aid of the funds of the Joint Council of the British Red Cross Society and the Order of St. John of Jerusalem, is to be held next Thursday. I wish every success to the fund, and only hope that the organisation, which became so efficient during the war, may be enabled to carry out equally good work in peace time.—(Signed) Edward, P."

### NO LONDON HOSPITAL SATURDAY IN 1920.

The decision of the Metropolitan Hospital Saturday Fund to join forces with the Red Cross and the League of Mercy for the sake of "Our Week" this year involves the intermission of Hospital Saturday in London for the first time for thirty years. The thirty-three local committees of the fund in the London district, which organise the work of half-a-million supporters, have been placed in touch with the divisional secretaries of the Red Cross Society. Meantime the Saturday Fund is making progress, and has received £10,000 more than during the corresponding period last year. Mr. Philip Inman considers that this has come from the working classes, who have responded to the hospitals' need, and not from the new rich, who, in many cases, are still uneducated to the duties of their new position. Hospital Saturday in the provinces is, of course, unaffected by the decision of the Metropolitan Fund, and the numerous local Saturday funds will make their own arrangements with the representatives of the Red Cross Society in their districts.

### HOSPITALS AND V.A.D.'s.

A Conference at Salisbury of representatives of Voluntary Aid organisations in Wiltshire, Hampshire, and Dorset discussed the future, after addresses from Sir Arthur Stanley and Sir Napier Burnett. Sir Harry Crichton, Chairman of the Hants County Territorial Association, said that, pending the War Office decision, the relation of the Association to the Hants branch of the V.A.D. should be the same as in pre-war days. The closer the two were brought together the better. He hoped that hospitals would welcome V.A.D. girls. Sir Arthur Stanley replied that a committee was considering the point. So far as he knew, the report strongly favoured the plan of keeping the V.A.D.s part of the Territorial Force, and making them a more intimate part than they were before. Colonel Twiss declared that it was now hard to get voluntary workers. Those who had worked during the war felt they had done their share. Good propagandists were needed. We should be interested to know how far the Committee thinks V.A.D.s can be useful to hospitals, and what are their recommendations in this matter.

### THE NATIONAL RELIEF FUND GRANT TO HOSPITALS.

The Chief Secretary for Ireland has informed the Irish Public Health Council that a substantial share of the £700,000 which is being allocated to voluntary hospitals by the National Relief Fund will be granted to Irish hospitals under certain definite principles, which will be applicable to the hospitals elsewhere in the United Kingdom. In respect of a request that State grants for public-health services should be continued, notwithstanding the refusal of public-health authorities to submit their accounts to audit, the Chief Secretary was unable to make an equally favourable announcement; it being hardly to be expected, in his opinion, that public moneys can be entrusted to local bodies who take up this position. He informed the Council that the real appeal lies to the local bodies, which prefer to sacrifice the health and lives of the children, the poor, and the afflicted, rather than submit to reasonable and legal conditions as to the examination of their accounts.

### THE MYSTERIOUS VISITOR.

One afternoon last week a gentleman who is described as of "unpretentious dress and demeanour" called at the Royal National Orthopædic Hospital, Great Portland Street, and handed Mr. Morley, the Secretary, a bank-note for £500, as a donation for the general welfare of the patients. He gave no name and desired that no receipt should be issued to him. He then departed with good wishes to the hospital, the whole duration of his visit having been only two or three minutes. It was presumably the same donor who on Friday last week called at two hospitals in another part of London and distributed a bank-note for the same amount to each. At one of these hospitals he was more communicative and said that he was seventy-five years of age and did not expect to have much more need for his money. He therefore wished to place it to as good use as possible and thought that the hospitals of London were as deserving of help as any other objects. This method of generous charity is rare in these days, although within the recollection of many hospital workers the anonymous caller bringing substantial contributions was not infrequent.

### M.P.'S ON THE HOSPITAL QUESTION.

No doubt with the laudable desire to focus public attention upon hospital needs before Red Cross Week and Our Day brings the opportunity to help before their readers, the newspapers have again been printing articles on the hospital problem. Several of these articles have been by Members of Parliament, but we are bound to confess that the writers have little of interest to say. A Labour member, however, appeals to the building trade to make an exception in favour of hospitals, to insure uninterrupted work, and, if possible, to speed up production, in the interests of the workers, who form the majority of cases on the waiting lists. Sickness, he adds, loses thousands of pounds to the workers where prompt treatment is not forthcoming. Though vaguely in favour of a State medical service, this Labour representative approves of the leagues of subscribers lately formed in the North. In truth, Labour is increasingly generous to hospitals.

### FLAG-DAYS IN LEEDS.

The Leeds hospitals, which would chiefly benefit by the proposed flag-day in connection with the campaign of the British Red Cross Society and the Order of St. John of Jerusalem, are naturally disappointed by the refusal of the Leeds Watch Committee to agree to the application to organise a flag-day. The Watch Committee explain that they can grant in no case permission to societies " who did not enjoy the privilege prior to the outbreak of war. This appeals to us as extremely unfair; to exercise proper precautions in this matter is quite as it should be, but to set up a system of vested interests in the direction of collecting for charities is a step which cannot be defended. The Red Cross movement requires the support of everybody. It is a great movement with great responsibilities, and is therefore one which in every detail must be organised in a way which will not be open to disagreeable features which are clear enough in respect of many flag-days and similar functions. It is quite easy to give a guarantee to the Leeds Watch Committee which will answer all objections, and we trust that this will be arranged.

### "LEAVINGS" FOR THE HOSPITALS.

It is to be hoped that the following experience of a certain clergyman is not typical of the Church's charity as a whole. The said clergyman, who has had some ten years' work in various parishes, complains that unless a special stir or effort is made by those interested, local hospitals generally occupy a second place—or third if there is one—in the charitable distributions of the Church; for example, after a Harvest Festival the gifts sent are allocated first to the sick and poor of the parish, and then, after careful inquiry from district visitors, etc., etc., for other "deserving cases." What is not required is sent off to the local hospital. It is perfectly right that the sick of the parish should receive gifts, but it seems a somewhat selfish attitude to retain the best and give to the local institution merely the "leavings." To say the least of it, such action creates the impression either that hospitals do not require help, or that "anything is good enough" for them—both ideas very erroneous and out of all harmony with the professed teaching of the Church.

### A STRIKE ON A HOSPITAL CONTRACT.

The painting of Queen Mary's Hospital, apparently the institution at Roehampton, has been interrupted by a strike of the workmen. Fortunately it was the firm which employed them, Messrs. A. A. Secrete, and not any action on the part of the hospital, which occasioned the trouble. It is stated that the firm placed a number of labourers on the preparation of woodwork, to which the organised trade unionists objected; that the firm consented to place the work with the painters, but discharged the man who reported the matter to his Union. A Union official visited the work, but a settlement was not made, he says, because the firm declined to call off certain labourers from painting certain ironwork which is considered part of the painting trade. The twenty-five painters were therefore instructed by the Union official, perhaps too hastily, to leave the job, and the vacant places were filled by men from the local Labour Exchange. The technical points in dispute are under consideration by the official representatives of the Master Builders and of the operatives, and on these we offer no opinion. But the painting of a hospital is a matter that everyone desires to see finished quickly, especially the patients, to whom the smell of paint, if unnecessarily prolonged, is a serious aggravation of their troubles.

### STREET MARKETS AND FOOD CONTAMINATION.

The growth of street markets in the Metropolis since the cessation of the war has been phenomenal, and it was advisable that some steps should be taken more adequately to supervise them and remove any of the many opportunities that arise through foodstuffs being contaminated. Many of the goods offered for sale on the stalls of street markets are more liable to contamination than those offered for sale in a shop. They are often allowed to stand near the kerb unprotected until such time as the goods on the stall have been cleared. Nor are only stallholders to blame, for many butchers and fishmongers have open boards in front of their shops only a short distance away from the pavement. The London County Council are asking whether the borough councils would be prepared to support them in obtaining legislation that would empower them to exercise a more thorough supervision over foodstuffs offered for sale in street markets, and general consent has been given to the proposal. This being the case, the London County Council propose in their next General Powers Bill to ask Parliament to sanction a number of clauses that will give them power to inspect and stop the contamination of food in street markets.

### ANNUAL HELP FROM LOCAL MAYORS.

Lieut.-Colonel Sir W. A. Wayland, who has been Mayor of Deptford for the past six years, lately reviewed the work done for the Miller General Hospital appeal at a dinner to those associated with him in the effort. Though the sum of £16,000 was aimed at, as a memorial to those who fell, the total collected was little more than £6,000. The Mayor therefore urged his successors in office to undertake to raise £3,000 a year to build, equip and endow a children's ward at the hospital. It was really a Deptford institution from which came sixty or over sixty per cent. of the cases, though situated in Greenwich. By direct contributions he thought perhaps £1,000 a year could be raised, while an Old English Fair at the Goldsmiths' College was an attraction which never failed. The children's ward would be as fine a memorial of the fallen as could be devised. Other speakers urged that successive mayors should unite to raise the remaining £10,000, a form of "municipal help" which the long term of office of the present Mayor might well induce to become traditional. Experience is showing that it is easier nowadays to raise yearly contributions than big sums; and enthusiastic mayors by successive efforts might give invaluable help.

### THE DUTIES OF SANATORIUM GARDENERS.

The Keighley and Bingley Joint Hospital Ward has lately dismissed three gardeners, who were in its employ at the hospital and sanatorium, in consequence of "the deplorable state of the grounds." The gardeners, in a very well-written letter, protest against the suggestion that they have neglected their duties. They say that the military authorities, who occupied the institution for three years, practically let the place run wild, because the one gardener was kept busy disinfecting clothes and driving an ambulance; that without extra help the grounds could not be restored in the interval; that they have been employed to remove tons of accumulated rubbish, and to drive the ambulance as well. They then describe the duties which this hospital's gardener is expected to perform: To feed and clean seven pigs at the sanatorium; to feed and rear sixty chickens and truss those required for the table; to carry coal to each ward; to screen ashes and refuse; and, lastly, to attend to the garden. In short, they say that "a gardener at the hospital has to be a handy man all round, responsible for duties too numerous to mention." Messrs. Shoesmith, Moorhouse, and Fox protest, therefore, that their character has been questioned unfairly, especially since the public has not been told the extent of the duties, the arrears of the work, and the fact that one of the gardeners, all of whom are ex-Service men, has been in the Board's service for twenty-four years. It will appear then that the gardeners do not deny the bad state of the grounds, but seek to explain it. They have, at all events, made two points: first, that the state of the grounds itself is not sufficient to justify dismissal, unless the interval since the military departed can be proved to have been misspent; and, secondly, that the duties need stricter definition, for variety causes much unavoidable waste of time, and ambulance work necessitates absences.

### A CENTURY AND A HALF OF SILENT WORK.

Not to have turned to the public for help in one hundred and fifty-five years is a proud claim for any hospital, but it is made by the General Lying-in Hospital, which is now appealing, apparently, for the first time since its foundation in 1765. The present building is much too small, and the Ecclesiastical Commissioners and the Rolls Estate have made a convenient offer of an open site near Walworth Road, in the centre of a crowded district. To build and equip a new hospital £100,000 is required, and the King's Fund has given its blessing to the scheme by a grant of £3,000 towards it. Among other things, the hospital is an established training-school for midwives and nurses; and we hope that the appeal, if only because of its exceptional circumstances, will make its claim heard among the rest.

### THE NORTH MIDDLESEX HOSPITAL.

The Edmonton Infirmary, with the consent of the Ministry of Health, has changed its name, to become known as the "North Middlesex Hospital." The Medical Superintendent is Dr. Spencer Mort, who became Colonel-in-Charge when the infirmary was used as a military hospital during the war. The Matron, Miss A. Dowbiggin, is also retaining her post. The guardians are proud of their institution—*x*-ray apparatus and radiant heat baths—and of their maternity wards, where midwives are trained. They hope that the newly christened hospital will soon convince the general public that it can do as much for those in need as it did for several thousand soldier patients.

### THIS WEEK'S DRUG MARKET.

Buying continues to be restricted to immediate requirements, and the volume of business is still very small. An advance in the price of glycerin has been announced; the increase is at the rate of about seven per cent., and was not altogether unexpected. Most of the price changes, however, are in a downward direction; they include a reduction in the quotation for codeine, but no change has been made in the price of morphine. Menthol and Japanese peppermint oil are again rather lower; camphor is unchanged. Quinine has a lower price tendency. In synthetic products there are no changes of note.

